# Proteo‐Metabolomic Profiling of PMM2‐CDG Reveals Dysregulation of Retinoic Acid Synthesis, *Myo*‐Inositol, and the Hexosamine Pathway

**DOI:** 10.1002/jimd.70233

**Published:** 2026-08-13

**Authors:** Diana Gallego, Giuseppina Andreotti, Maria Monticelli, Debora Paris, Arturo Martín‐Martínez, Alejandra Gámez, Mercedes Serrano, José Córdoba‐Caballero, Pedro Seoane, Juan A. G. Ranea, Belén Pérez

**Affiliations:** ^1^ Centro de Diagnóstico de Enfermedades Moleculares. Centro de Biología Molecular Universidad Autónoma de Madrid. CIBERER. IdiPAZ Madrid Spain; ^2^ Institute of Biomolecular Chemistry (ICB) National Research Council of Italy Naples Italy; ^3^ UniCamillus ‐ Saint Camillus International University of Health Sciences Rome Italy; ^4^ Pediatric Neurology Department, Hospital Sant Joan de Déu Barcelona, Spain and Institut de Recerca Sant Joan de Déu, CIBERER Barcelona Spain; ^5^ Department of Molecular Biology and Biochemistry, University of Malaga, 29010, Malaga, Spain. CIBERER. Institute of Biomedical Research in Malaga and Platform of Nanomedicine, IBIMA Plataforma BIONAND, 29071, Malaga, Spain. Spanish National Bioinformatics Institute (INB/ELIXIR‐ES) Instituto de Salud Carlos III (ISCIII) Madrid Spain

**Keywords:** hexosamine pathway, metabolomics, *myo*‐inositol, PMM2‐CDG, proteomics, retinoic acid, retinol

## Abstract

Phosphomannomutase deficiency (PMM2‐CDG), the most common congenital disorder of glycosylation (CDG), is characterized by multisystem involvement and a lack of disease‐modifying therapies. While previous transcriptomic studies have uncovered disrupted cellular pathways, the functional consequences of these alterations remain poorly understood. To further investigate PMM2‐CDG pathophysiology, we integrated proteomic and metabolomic profiling of patient‐derived fibroblasts with previously published transcriptomic data. Proteomic analysis was performed using Tandem Mass Tag‐based mass spectrometry, while metabolomics was conducted via Nuclear Magnetic Resonance spectroscopy. Multi‐omics integration was performed using principal component analysis–based dimensionality reduction, incorporating clinical metadata as supplementary variables. Proteomic analysis identified 43 significantly altered proteins, with enrichment in the retinoic acid synthesis pathway, wound healing, and cytoskeletal organization. Metabolomic profiling revealed altered amino acid levels and elevated concentrations of UDP‐GlcNAc, consistent with perturbation of the hexosamine biosynthesis pathway, and increased levels of *my* *o*‐inositol. Notably, *myo*‐inositol levels showed a strong association with disease severity in the integrated analysis. RT‐qPCR confirmed the upregulation of *GFPT2*. This integrative multi‐omics study identifies consistent alterations in the retinoic acid synthesis pathway and hexosamine biosynthesis in PMM2‐CDG patient‐derived fibroblasts and reveals an association between intracellular *myo*‐inositol levels and disease severity. These findings provide new insights into PMM2‐CDG–associated molecular alterations and illustrate the value of multi‐omics integration for hypothesis generation in rare diseases.

## 1 Introduction

Congenital disorders of glycosylation (CDG) are a diverse group of rare metabolic diseases characterized by defects in the glycosylation of proteins and lipids, a fundamental process for normal cellular function [1]. Among them, PMM2‐CDG is the most prevalent subtype, caused by a deficiency of the enzyme phosphomannomutase 2 (PMM2). This enzyme catalyzes the conversion of mannose‐6‐phosphate to mannose‐1‐phosphate, an essential step in the N‐glycosylation pathway, by using glucose‐1,6‐bisphosphate (Glc‐1,6P_2_) as an activator. Other than crucial for catalysis, Glc‐1,6P_2_ also acts as a stabilizer [2]. PMM2‐CDG is a multisystemic and often life‐threatening disorder, with predominant neurological involvement but also affecting other organ systems [3].

Current treatment options remain largely supportive and symptomatic. Disease‐modifying strategies under investigation include pharmacological chaperones and substrate replacement approaches aimed at restoring glycosylation [4, 5, 6, 7]; however, their efficacy is variable and incomplete, underscoring the need for a deeper understanding of disease mechanisms to enable the development of more effective therapies [8, 9, 10].

Although defective glycosylation is the primary biochemical hallmark of PMM2‐CDG, the mechanisms linking hypoglycosylation to downstream cellular dysfunction and organ pathology remain incompletely understood. Glycosylation defects have been shown to disrupt protein folding, trafficking, and receptor signaling, leading to activation of stress responses such as the unfolded protein response and alterations in endoplasmic reticulum and Golgi homeostasis [11]. Recent work in PMM2‐deficient human neural models has revealed both neural and metabolic dysregulation resulting from impaired N‐glycosylation, providing mechanistic insight into the neurological manifestations of PMM2‐CDG [12]. In addition, altered glycosylation of cell surface receptors has been shown to modulate immune signaling, including tumor necrosis factor–related pathways, suggesting that immune and inflammatory responses may contribute to PMM2‐CDG pathophysiology [13]. Moreover, using a zebrafish model of PMM2 deficiency, impaired N‐glycosylation was shown to alter hexosamine pathway activity and increase O‐GlcNAcylation, revealing functional cross‐talk between glycosylation pathways and cellular metabolism that may influence disease severity [14].

Beyond these primary effects, PMM2 deficiency has been associated with broader alterations in cellular organization and metabolism. Transcriptomic studies across CDG models have reported dysregulation of pathways related to extracellular matrix organization, cell adhesion, stress responses, and metabolic regulation, underscoring the systemic consequences of impaired glycoprotein function [15, 16].

In this context, we previously performed transcriptomic profiling of skin fibroblasts derived from PMM2‐CDG patients [17]. This analysis identified dysregulation of pathways related to cell cycle regulation, proliferation, extracellular matrix composition, and prostaglandin‐related signaling. Systems biology analysis using integrative bioinformatic approaches further highlighted alterations in cell senescence, bone‐related pathways, cell adhesion, extracellular matrix organization, and cytokine‐associated signaling. Importantly, pharmacological chaperone treatment partially reversed specific transcriptional alterations, supporting the therapeutic relevance of targeting defined molecular pathways.

While transcriptomics provides valuable insights into gene expression, it does not fully reflect functional protein levels or the metabolic state of the cells. Therefore, a more comprehensive understanding of disease mechanisms requires multi‐omics approaches, integrating data across the transcriptome, proteome, and metabolome [18]. Transcriptomics enables the quantification of mRNA levels and the identification of splicing variants and regulatory elements [19, 20], but proteomics offers direct insight into cellular function by measuring protein abundance and post‐translational modifications such as glycosylation, phosphorylation, and ubiquitination [21, 22]. In parallel, metabolomics quantifies small molecules such as amino acids, sugars, and lipids, providing a dynamic snapshot of cellular metabolism and serving as a functional readout of disease processes [18, 23].

Integrating these layers of biological information allows for an integrative, pathway‐level understanding of PMM2‐CDG pathophysiology by linking gene expression changes with corresponding protein and metabolic alterations. Such strategies can uncover novel molecular biomarkers and therapeutic targets, as demonstrated in previous omics‐based studies in CDG and other metabolic disorders [9, 14, 24]. Tools such as principal component analysis (PCA) enable the integration of diverse omics data types—such as transcriptomics and metabolomics—by identifying shared patterns that explain variance across datasets [17].

In this study, we expanded upon our previous transcriptomic analysis by incorporating proteomic and metabolomic profiling of fibroblasts derived from PMM2‐CDG patients. This multi‐omics approach revealed novel dysregulated pathways, including the retinoic acid synthesis pathway and the hexosamine biosynthetic pathway, and uncovered significant changes in key intracellular metabolites, such as UDP‐GlcNAc, amino acids, and *myo*‐inositol. Notably, the integration of molecular and clinical data revealed an association between intracellular *myo*‐inositol levels and disease severity. Together, our findings offer a more comprehensive view of PMM2‐CDG pathophysiology and provide new leads for future mechanistic and therapeutic studies.

## 2 Materials and Methods

### 2.1 Patient and Sample Collection

The patient cohort, clinical assessments, and sample collection procedures were as described previously in [17] and included in Table S1. Briefly, fibroblast cell lines were established from 12 PMM2‐CDG patients (9 males, 3 females) carrying pathogenic variants with potential responsiveness to pharmacological chaperones (PCs). Patients were clinically evaluated using the Nijmegen Pediatric CDG Rating Score (NPCRS), the International Cooperative Ataxia Rating Scale (ICARS) [25, 26], and the midsagittal vermis relative diameter (MVRD) measured by MRI [27]. Findings were annotated with Human Phenotype Ontology (HPO) terms. Additional clinical and molecular details are available in [17]. Fibroblasts from six healthy individuals were used as control cell lines. We had amino acid quantification data from 23 PMM2‐CDG patients, two of whom were also included in the metabolomics analysis.

Both patient‐ and control‐derived fibroblasts were maintained in minimal essential medium (MEM) supplemented with 1% L‐glutamine, 10% fetal calf serum (FCS), and antibiotics. For RNA and proteomic experiments, cells were transiently synchronized to reduce cell‐cycle–related variability by serum deprivation (MEM with 0.5% FCS and 1% glutamine) for 48 h, followed by 24 h in complete medium before collection. This approach was used to facilitate differential gene and protein expression analyses while minimizing variability arising from differences in cell‐cycle state. No synchronization or nutrient deprivation was applied for metabolomic experiments to avoid perturbation‐sensitive metabolic pathways.

### 2.2 Proteomic Analysis of Fibroblasts

Proteomic profiling was performed on fibroblasts from three PMM2‐CDG patients and three healthy controls (Table S1). Cells were seeded in 100 mm dishes (2 million cells/plate), subjected to the synchronization protocol described above, and lysed using a buffer containing 150 mM NaCl, 1% Triton X‐100, 0.5% sodium deoxycholate, 0.1% SDS, and 50 mM Tris–HCl (pH 7.4), supplemented with EDTA‐free protease inhibitors (Roche). Lysates were subjected to nitrogen freeze–thaw cycles and homogenized through a 22G needle. Insoluble material was removed by centrifugation.

Protein digestion was performed with sequencing‐grade trypsin (Promega) at a 1:20 (w/w) enzyme‐to‐substrate ratio overnight at 37°C. Peptides (100 μg/sample) were labelled with the TMT 6‐plex Isobaric Mass Tagging Kit (Thermo Fisher Scientific) and analysed by reverse‐phase LC–MS/MS using an Easy‐nLC II system coupled to an LTQ‐Orbitrap Velos Pro hybrid mass spectrometer.

Peptides were identified and quantified using PEAKS Studio X Pro (Bioinformatics Solutions Inc.) against the *Homo sapiens* Uniprot database. Results were filtered at 1% FDR. Differentially abundant proteins (*p* < 0.05, FDR < 0.01) were analyzed for Gene Ontology Biological Process enrichment using the ClusterProfiler package in R (v4.3.1) [28].

### 2.3 Metabolomic Analysis of Fibroblasts

Fibroblasts from twelve PMM2‐CDG patients and six healthy controls (Table S1) were seeded in 150 mm culture dishes and grown under standard conditions (MEM supplemented with 1% L‐glutamine, 10% FCS, and antibiotics). Cells were maintained until reaching near confluence (approximately 80%–90%) and harvested by trypsinization. Medium was refreshed every two days during culture. At the time of harvesting, cells were counted to ensure that comparable cell numbers (approximately 10 million cells per line) were processed for metabolomic analysis. Cell pellets were flash‐frozen in liquid nitrogen. Metabolites were extracted using a methanol–chloroform–water protocol [29], separating polar and apolar phases. Only the polar phase yielded quantifiable NMR data and was used for subsequent analyses.

Samples were dissolved in PBS containing 0.1 mM trimethylsilylpropanoic acid (TSP). NMR spectra were acquired using a Bruker Avance III 600 MHz spectrometer with a TCI CryoProbe and Z‐gradient (Bruker BioSpin GmbH) at 27°C, following the method described by [5]. One‐dimensional (1D) ^1H NMR and two‐dimensional (2D) TOCSY and HSQC spectra were acquired. Metabolites were identified by matching chemical shifts with published references [30] and databases [31].

Spectra were binned using AMIX 3.9.7 (Bruker), and the resulting data matrices were analysed in SIMCA 14 (Umetrics). Unsupervised PCA was used to assess data structure and identify outliers. Subsequently, supervised Orthogonal Partial Least Squares Discriminant Analysis (OPLS‐DA) was performed to discriminate between groups. Model performance was evaluated using R^2^ (78%), Q^2^ (51%), and validated by 7‐round permutation testing (800 iterations) and CV‐ANOVA (*p* = 0.03).

Metabolites with correlation loadings |p(corr)| > 0.7 were considered significant and subjected to further statistical analysis. Group differences (patients vs. controls) were tested with limma (moderated t; contrast patient–control), and multiplicity was controlled using Benjamini–Hochberg FDR. Table S4 reports raw data, bins, and their related groups for the metabolite assignment, and for each analyzed feature, the log2 fold‐change, fold‐change, *p*‐value, FDR, and t‐statistic. Statistical analyses were conducted using OriginPro 9.1 (OriginLab) and R (R Core Team, 2021).

### 2.4 RT‐qPCR Analysis

To validate gene expression changes, total RNA was extracted and reverse‐transcribed into cDNA using the NZY First‐Strand cDNA Synthesis Kit (Nzytech) from 500 ng of RNA. Quantitative PCR was performed with PerfeCTa SYBR Green FastMix (Quanta Biosciences) on a Bio‐Rad CFX Opus 384 system. Gene expression was normalized to *GUSB*, and relative fold changes were calculated using the 2^−ΔΔCt method. Data analysis was performed using GraphPad Prism, version 8, and statistical comparisons between groups utilized an unpaired *t*‐test with Welch's correction. Primers sequences used in this study are listed in Table S2.

### 2.5 Western Blot Analysis

To validate changes in protein levels, fibroblasts were cultured under standard conditions without synchronization prior to protein extraction. Total protein was extracted from cell/tissue pellets using RIPA buffer (with Protease Inhibitor Cocktail, Roche) and quantified via the Bradford Protein Assay Kit (Bio‐Rad). 10 μg of protein were resolved by SDS‐PAGE using NuPAGE Bis‐Tris precast gels (4%–12% gradient, Thermo Fisher Scientific). Proteins were then transferred to a nitrocellulose membrane (Invitrogen) using the iBlot 2 device and Regular Stacks (Thermo Fisher Scientific).

Membranes were blocked in 5% nonfat dry milk in TBS‐T (0.1% Tween 20) and incubated overnight at 4°C with primary antibodies: Anti‐ALDH1A1 (Rabbit polyclonal, 1:1000), Anti‐ALDH1A3 (Rabbit polyclonal, 1:1000), and Anti‐GAPDH (Mouse monoclonal, 1:5000). Following washing, HRP‐conjugated secondary antibodies (Goat anti‐Rabbit‐HRP, 1:5000; Horse anti‐Mouse‐HRP, 1:2000) were applied for 1 h at room temperature. Bands were visualized using SuperSignal West Femto (Thermo Fisher Scientific).

Densitometry was performed using Fiji ImageJ. Protein expression was normalized to GAPDH, and relative expression levels were calculated from the ratio of target band intensity to GAPDH intensity. Data analysis was performed using GraphPad Prism, version 8, and statistical comparisons between groups utilized an unpaired *t*‐test with Welch's correction.

### 2.6 Measurement of Plasma Amino Acids

Plasma samples from 23 PMM2‐CDG patients were used for amino acid quantification. A panel of 42 amino acids was measured using ion exchange chromatography. Amino acid quantification was performed using 200 μL of plasma from PMM2‐CDG patients. Proteins were precipitated by adding 50% sulfosalicylic acid. Samples were thoroughly mixed using a vortex mixer and incubated on ice for 30 min, followed by centrifugation. Before analysis, the resulting supernatant was diluted 1:4 with injection buffer. Amino acid analysis was carried out using a Biochrom 30+ amino acid analyzer. Plasma amino acid concentrations were interpreted using age‐specific reference intervals routinely employed for clinical diagnosis. Because amino acid concentrations vary substantially during childhood, pediatric reference intervals were stratified into the following age groups: 0–3 months, 3 months–1 year, 1–3 years, 3–10 years, and > 10 years.

To validate glutamine levels in fibroblasts and conditioned culture medium, control and PMM2‐CDG fibroblasts were cultured under standard conditions and samples were analyzed by ion‐exchange chromatography using the same amino acid analyzer. Intracellular glutamine concentrations were normalized to total protein concentration, whereas glutamine levels in conditioned medium were normalized to cell number.

### 2.7 Integration of Transcriptomic and Metabolomic Data

Integration of transcriptomic and metabolomic datasets was performed using principal component analysis (PCA). The analysis included 10 patients from the cohort of 12 PMM2‐CDG patients used in the omics studies. P7 was excluded due to a lack of comprehensive clinical data. P6 was also excluded because it was the only patient with an intermediate clinical phenotype and could not be confidently assigned to either the mild or severe group used for the severity‐based differential expression analysis. Gene expression data (normalized counts from [17]) were filtered to retain 25 differentially expressed genes using the Exp Hunter Suite [32]. Supplementary variables included clinical scores (age, NCPRS, ICARS, and MVRD) and metabolite data from the current study; however, only the significant differential metabolites for control‐ disease comparison were retained.

PCA was applied to gene expression data to transform correlated variables (gene expressions) into PCs through linear transformation. The PCA and the relevant component selections were done using the FactoMineR package [33]. Once the PCA was finished we applied (1) a statistical test over the relation between Principal Component (PC) and external data from samples and (2) a Hierarchical Clustering on Principal Components (HCPC) using the relevant PCs from gene expression data. We projected the metabolomic data, age, and disease severity scales (also known as external variables) to PCA space using the methodology of Lê and collaborators [33] without affecting the generated PCs. In this case, we applied the pre‐computed “transition formulae” that are used to transform the raw data to PC space coordinates and then we calculated for each external variable its Pearson correlation coefficient with the individuals' coordinates on the PCs [33]. In relation to HCPC, the hierarchical tree was computed using Ward's method [34] and the number of clusters was automatically calculated based on the increase of inertia that measures the proportion of change from one partition to another and selects the best optimal [35]. Supplementary variables significantly correlated with principal components were reported.

### 2.8 AI And AI‐Assisted Technologies in the Writing Process

During the preparation of this work, the author(s) used Grammarly and ChatGPT 5.2 to assist with English language editing and clarity. All content generated or modified using these tools was critically reviewed and edited by the author(s), who take full responsibility for the final content of the manuscript.

## 3 Results

### 3.1 Proteomic Analysis of PMM2‐CDG Fibroblasts Reveals Affected Cellular Pathways

In our previous study, RNA‐seq analysis of skin fibroblasts from an initial cohort of three PMM2‐CDG patients and three healthy controls identified transcriptomic alterations affecting multiple cellular pathways, including processes related to cell proliferation, extracellular matrix composition, and prostaglandin synthesis. These findings were subsequently validated in an expanded cohort of five controls and twelve patients, confirming the robustness of the molecular signatures identified [17]. To determine whether these transcriptomic alterations were also reflected at the protein level and to explore additional molecular changes that may not be evident from transcriptomic data alone, we performed proteomic analysis on fibroblasts derived from the same initial cohort (P6, P7, and P9 and three healthy controls). Protein quantification was carried out using mass spectrometry to identify differentially abundant proteins.

**FIGURE 1:**
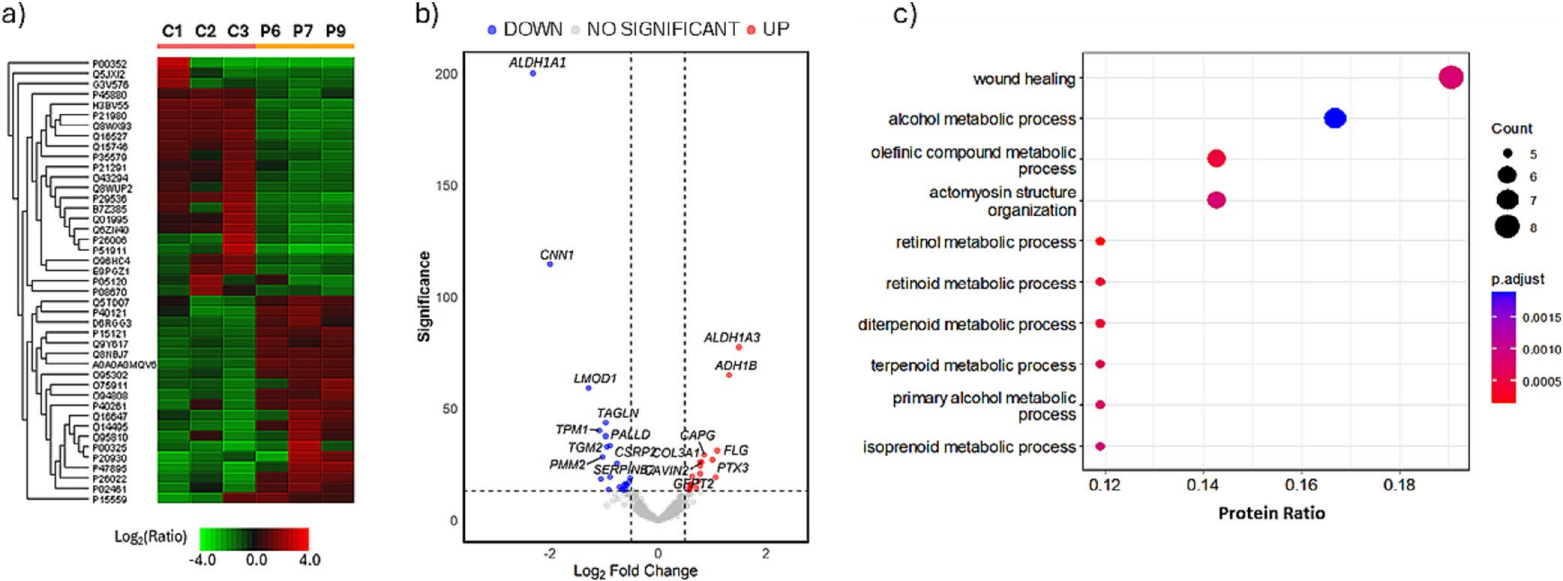
Proteomic analysis reveals dysregulation of enzymes involved in retinoic acid metabolism in PMM2‐CDG patient fibroblasts. (A) Hierarchical clustering heatmap of differentially expressed proteins in fibroblasts from PMM2‐CDG patients (P6, P7, P9) versus healthy controls (C1–C3), based on log_2_‐transformed expression ratios. Color scale indicates protein abundance: Green = downregulated, red = upregulated. (B) Volcano plot showing differentially expressed proteins. X‐axis: Log_2_ fold change; Y‐axis: Significance level (−log_10_ *p*‐value). Proteins in red are significantly upregulated, blue are significantly downregulated, and grey represent non‐significant changes. Key enzymes in the retinoid pathway are highlighted. (C) Functional enrichment analysis of dysregulated proteins in PMM2‐CDG fibroblasts. Dot plot representing enriched Gene Ontology (GO) biological processes among the differentially expressed proteins identified in patient‐derived fibroblasts. Each dot corresponds to an enriched term, with X‐axis representing the gene ratio (number of significant proteins associated with a given term divided by total number of proteins in the term), dot size indicating the number of proteins involved (Count), and dot color corresponding to the adjusted *p*‐value (p.adjust), with red indicating the most statistically significant terms.

A total of 43 proteins exhibited statistically significant abundance changes in PMM2‐CDG fibroblasts relative to controls (Table S3), with 23 downregulated and 20 upregulated (Figure 1). Key dysregulated proteins are highlighted in the volcano plot (Figure 1B). Among the downregulated proteins, we identified several involved in cytoskeletal organization and actin‐binding, including calponin‐1 (encoded by the gene *CNN1*), leiomodin‐1 (*LMOD1*), transgelin (*TAGLN*), tropomyosin (*TPM1*), protein‐glutamine gamma‐glutamyltransferase (*TGM2*), palladin (*PALLD*), and myosin‐9 (*MYH9*). As expected, PMM2 protein levels were also reduced.

In contrast, the upregulated proteins displayed more diverse functional roles. Notable examples include glutamine‐fructose‐6‐phosphate aminotransferase (encoded by the gene *GFPT2*), a key enzyme of the hexosamine pathway, as well as prostacyclin synthase (*PTGIS*) and pentraxin 3 (*PTX3*), both implicated in inflammatory responses. Notably, prostacyclin synthase is well‐known for its role in producing prostacyclin, a lipid mediator with prominent anti‐inflammatory and protective vascular effects [36]. Interestingly, pentraxin 3 had been previously highlighted as up‐regulated in the PMM2‐CDG mouse model [37], and its decrease had been highlighted as a marker of restored glycosylation in PMM2‐CDG fibroblasts following PMM1 knock‐out and Glc‐1,6‐P_2_ increase [5].

**FIGURE 2:**
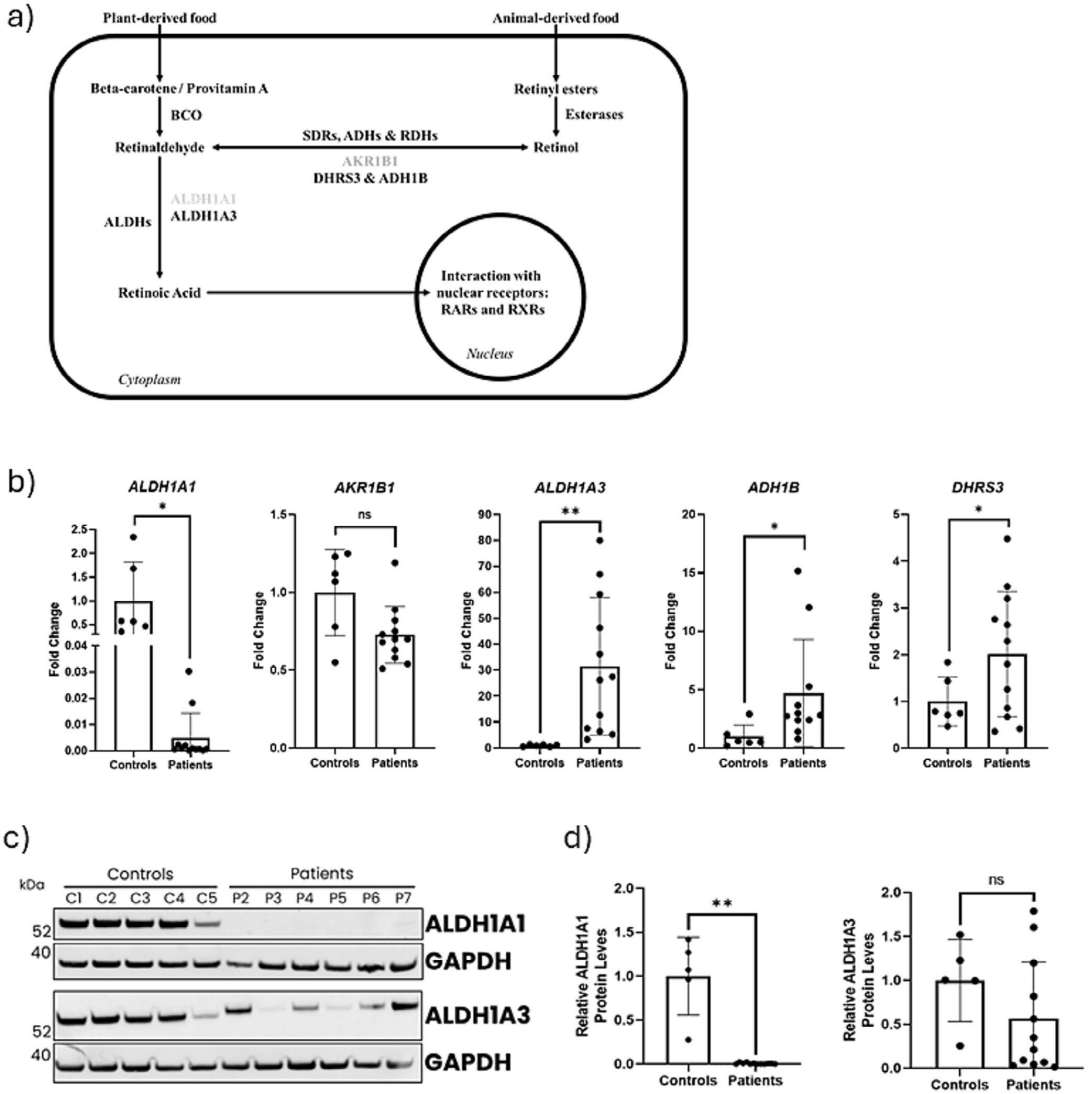
Relative expression of retinoic acid synthesis‐related genes in PMM2‐CDG patient‐derived fibroblasts. (A) Enzymatic pathways for the metabolism of retinoic acid. BCO: Beta‐carotene oxygenase, SDRs: Short‐chain dehydrogenases/reductases, ADHs: Alcohol dehydrogenases, RDHs: Retinol dehydrogenases, ALDHs: Aldehyde dehydrogenases. Retinoic acid activates genes through the retinoid X receptor (RXR) in regions called retinoic acid response elements (RAREs). (B) Bar graphs showing normalized mRNA abundance (fold change) of selected enzymes related to retinoic acid metabolism determined by RT‐qPCR analysis. (C) Immunodetection of ALDH1A1 and ALDH1A3 in 5 controls and 6 patients' fibroblast samples. The same amounts of total protein from the soluble extracts were loaded onto SDS‐PAGE gels. GAPDH was used as a loading control. (D) Densitometry of band sizes from the western blot of proteins ALDH1A1 and ALDH1A3 in 5 controls and 12 patients' fibroblast samples. Data are presented as mean ± SD. Statistical significance: *p* < 0.05 (*), *p* < 0.001 (**), ns = not significant.

To explore the biological relevance of these alterations, we performed Gene Ontology (GO) enrichment analysis. This analysis highlighted pathways related to metabolism and cytoskeletal regulation, particularly *wound healing* and *retinol metabolism* (Figure 1C). Proteins within these categories are summarized in Table 1. Wound healing proteins regulate actomyosin remodelling, influencing cell migration and proliferation. Retinol metabolism proteins are involved in retinoic acid synthesis, essential for cell differentiation and growth (Figure 2A).

**TABLE 1:** Functional terms enriched in the proteomic analysis of PMM2‐CDG fibroblasts.

GO term	Gene	Protein	Fold change	RNA‐seq log2 FC	Function	Clinical relevance in PMM2‐CDG
Wound healing	*PLPP3*	Phospholipid phosphatase 3	2.1	1.31	Wnt signaling pathway. Cell proliferation and differentiation	May contribute to vascular dysfunction, edema, and coagulopathy
	*COL3A1*	Collagen alpha‐1 (III) chain	1.71	Not DEG	Structure of connective tissue	Connective tissue weakness, joint hyperlaxity, poor wound healing, skeletal deformities
	*FGF2*	Fibroblast growth factor 2	1.68	Not DEG	Regulation of cell survival, division, differentiation, and migration	Neurodevelopmental delay, impaired tissue repair, growth retardation
	*TPM1*	Tropomyosin alpha‐1 chain	0.47	Not DEG	Implicated in stabilizing actin cytoskeleton filaments	Hypotonia, muscle weakness, motor delay
	*SERPINB2*	Plasminogen activator inhibitor 2	0.59	Not DEG	Inhibits urokinase‐type plasminogen activator	Coagulation defects, increased risk of thrombosis, and inflammation
	*MYLK*	Myosin light chain kinase	0.65	Not DEG	Regulates actin‐myosin interaction. Involved in the inflammatory response, cell motility, and morphology	Vascular instability, edema, and possible impact on muscle tone
	*CSRP1*	Cysteine and glycine‐rich protein 1	0.69	Not DEG	Could play a role in neuronal development	Impaired tissue development and repair
	*MYH9*	Myosin‐9	0.7	Not DEG	Role in cytokinesis, cell shape, cell motility, secretion, and capping	Muscle weakness, impaired cell migration, increased risk of infections
Retinol metabolic process	*ALDH1A3*	Aldehyde dehydrogenase 1A3	2.83	3.01	Formation of retinoic acid (role in cell proliferation and differentiation)	May impact craniofacial features, CNS development, and neurological features
	*ADH1B*	All‐trans‐retinol dehydrogenase	2.49	Not DEG	Oxidation of all‐trans‐retinol (retinoid metabolism)	
	*DHRS3*	Short‐chain dehydrogenase/reductase 3	1.63	Not DEG	Reduction of all‐trans‐retinal to all‐trans‐retinol (retinoid metabolism)	
	*AKR1B1*	Aldo‐keto reductase 1B1	1.52	Not DEG	Reduction of a wide variety of carbonyl‐containing compounds to their corresponding alcohols	
	*ALDH1A1*	Aldehyde dehydrogenase 1A1	0.2	−6.75	Oxidation of retinaldehyde into retinoic acid	

To further explore the retinoic acid synthesis pathway, we conducted RT‐qPCR on fibroblasts from PMM2‐CDG patients (Figure 2B). *ALDH1A1, ALDH1A3*, and *ADH1B* showed significantly altered transcript levels, aligning with proteomic data. Although *DHRS3* exhibited similar trends, its changes were not statistically significant. Interestingly, AKR1B1 protein levels were elevated, but its gene expression was slightly decreased.

To validate the RT‐qPCR results at the protein level, western blot analysis was performed for ALDH1A1 and ALDH1A3 proteins (Figures 2C,D and Figure S1). The ALDH1A1 protein band was clearly detectable in the control samples, but its levels were markedly decreased in all patient samples. This substantial reduction in ALDH1A1 protein is consistent with both transcriptomic and proteomic data. In contrast, ALDH1A3 protein levels showed high variability in patients' cell lines, which does not appear to be related to patient severity. These findings suggest that increased *ALDH1A3* transcript levels may not uniformly lead to protein accumulation across all individuals, although elevated ALDH1A3 protein was observed in a subset of patients.

### 3.2 NMR‐Based Metabolomics Identifies Altered Amino Acids and Metabolic Intermediates

**FIGURE 3:**
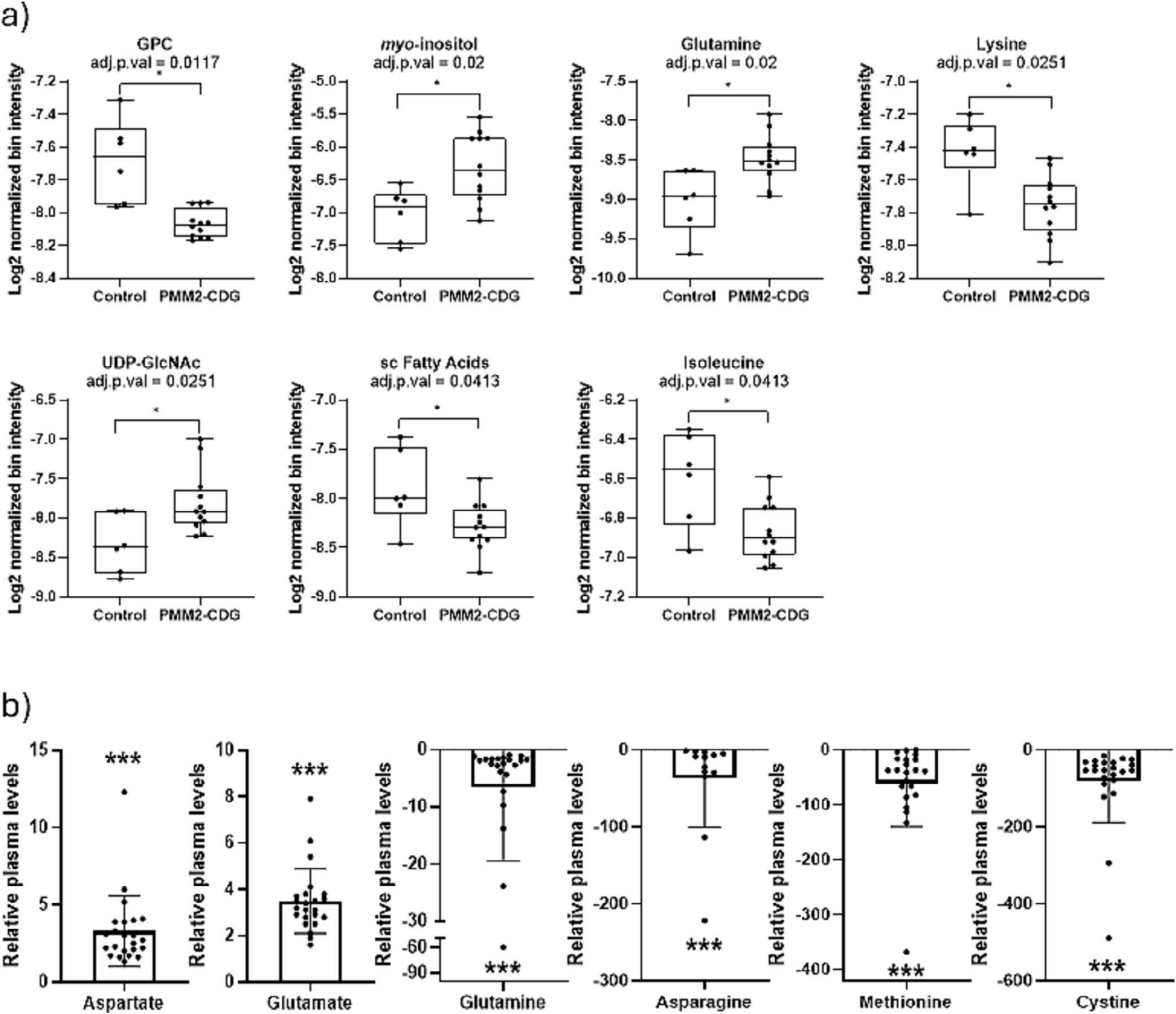
Polar metabolite alterations in PMM2‐CDG fibroblasts and plasma. (A) Boxplots showing significantly altered metabolites identified by ^1^H‐NMR metabolomics, expressed as Log2‐normalized bin intensities (Benjamini–Hochberg FDR *p* < 0.05). (B) Bar plots showing plasma levels of significantly altered amino acids in PMM2‐CDG patients expressed relative to age‐matched pediatric reference values. Data are presented as mean ± SD. *p < 0.05, ***p < 0.001.

Our prior transcriptomic and proteomic analyses pointed toward dysregulation of metabolic pathways. Given that PMM2‐CDG stems from defective mannose metabolism, we pursued an untargeted metabolomics study via NMR to quantify polar‐phase metabolites in fibroblasts from twelve PMM2‐CDG patients and six healthy controls to provide a comprehensive, untargeted snapshot of the metabolome [38]. All the significantly altered metabolites are reported in Figure 3A, together with their *p*‐values, while raw data for all the identified metabolites are reported as Table S4.

NMR analysis revealed moderate yet significant changes in amino acid profiles: glutamine was elevated, while isoleucine and lysine were reduced. Additionally, decreased levels of glycerophosphocholine (GPC) and short‐chain fatty acids were observed. Elevated levels of glucose metabolism intermediates were also detected, including *myo*‐inositol and UDP‐N‐acetylglucosamine (UDP‐GlcNAc), a sugar donor in N‐glycosylation.

Overall, glutamine, *myo*‐inositol, and UDP‐GlcNAc were identified as the most changed metabolites. These alterations are compatible with a perturbation of mannose‐1‐phosphate–dependent glycosylation pathways and associated hexosamine and glucose metabolism [39, 40, 41].

To complement fibroblast metabolomics, we performed plasma amino acid profiling in 23 PMM2‐CDG patients. Despite variability due to clinical heterogeneity, consistent alterations were detected (Figure 3B). Aspartate and glutamate levels were significantly elevated. Conversely, asparagine, methionine, and cystine were reduced. Notably, glutamine levels were decreased in plasma, contrasting with the elevated intracellular glutamine levels detected by NMR in fibroblasts.

To investigate whether intracellular glutamine accumulation could be explained by increased uptake from the extracellular medium, glutamine levels were measured by ion‐exchange chromatography in both fibroblast lysates and conditioned culture medium (Figure S2). Consistent with the NMR results, intracellular glutamine showed a trend toward increased levels in PMM2‐CDG fibroblasts. However, glutamine levels in conditioned medium were not significantly different between PMM2‐CDG and control cultures. These findings do not support increased glutamine uptake as the primary mechanism underlying intracellular glutamine accumulation.

**FIGURE 4:**
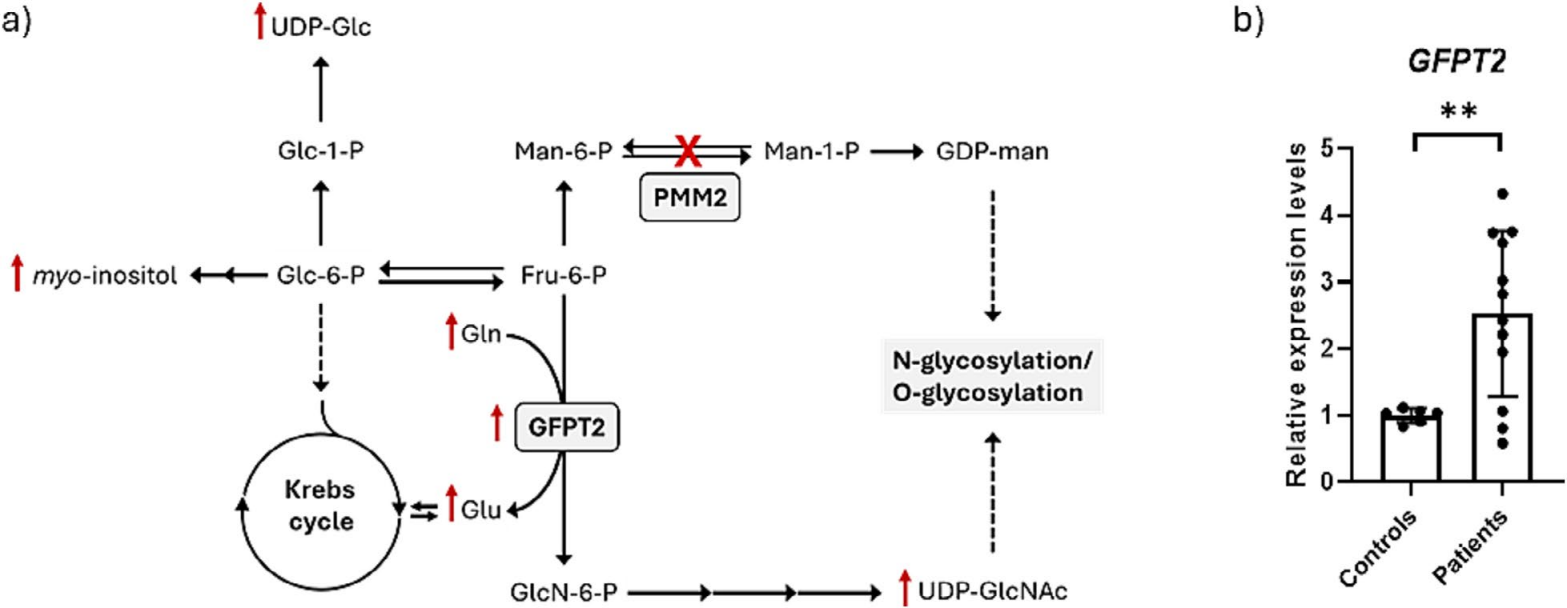
Dysregulation of the hexosamine biosynthetic pathway in PMM2‐CDG. (A) Schematic representation of metabolic flux involving glucose (Glc), mannose (Man), and glutamine (Gln) toward the synthesis of UDP‐GlcNAc via the hexosamine biosynthetic pathway. In PMM2‐CDG, the block in mannose‐6‐phosphate (Man‐6‐P) conversion to mannose‐1‐phosphate (Man‐1‐P) by PMM2 leads to redirection of flux toward fructose‐6‐phosphate (Fru‐6‐P). This results in increased activity of GFPT2, the rate‐limiting enzyme of the hexosamine pathway, producing more glucosamine‐6‐phosphate and UDP‐GlcNAc. PMM2, Phosphomannomutase 2; GFPT2, Glutamine–Fructose‐6‐Phosphate Aminotransferase 2; Glc, Glucose; UDP‐GlcNAc, UDP N‐acetylglucosamine. (B) Bar plot showing relative expression levels of *GFPT2* in fibroblasts from PMM2‐CDG patients compared to healthy controls determined by RT‐qPCR. Data are presented as mean ± SD. **p < 0.01.

In addition to these metabolite‐level changes, the increased abundance of UDP‐GlcNAc observed in PMM2‐CDG fibroblasts is consistent with activation of the hexosamine biosynthetic pathway. Supporting this, proteomic analysis identified upregulation of GFPT2, the inducible isoform of the enzyme catalyzing the first and rate‐limiting step of this pathway. RT‐qPCR analysis confirmed elevated *GFPT2* transcript levels in PMM2‐CDG fibroblasts compared to controls, although with marked inter‐individual variability (Figure 4B). Together, these findings indicate that increased UDP‐GlcNAc levels are associated with enhanced GFPT2 expression, suggesting that the hexosamine biosynthetic pathway is actively upregulated rather than reflecting passive accumulation of glycosylation donors due to impaired PMM2‐dependent N‐glycosylation.

### 3.3 Multi‐Omics Integration Highlights Clinical and Molecular Correlations

**FIGURE 5:**
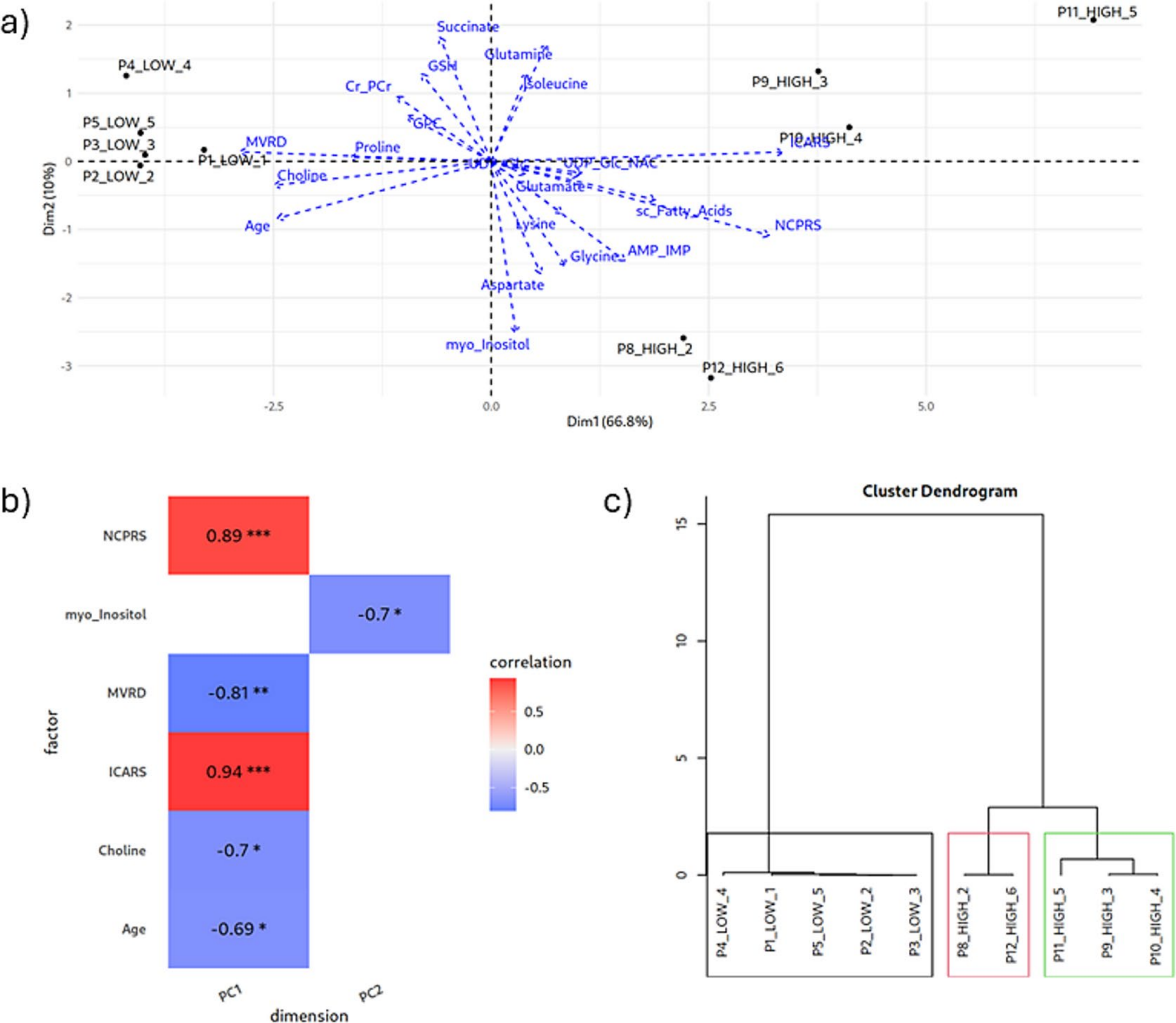
Representation of the metabolomic profiles and the clinical severity of the differentially expressed genes (DEGs) identified when mild and severe patients are compared. (A) Principal component analysis (PCA) biplot of DEGs identified between PMM2‐CDG patients with high clinical severity (“HIGH”) and low severity (“LOW”), showing separation between both groups. Arrows represent metabolite correlations, highlighting variables contributing to group separation, such as glutamine, UDP‐GlcNAc, glutamate, and aspartate. (B) Correlation heatmap between the first two principal components (PC1 and PC2) and clinical/biological factors. PC1 shows strong positive correlation with ICARS (ataxia severity; *r* = 0.94, *p* < 0.001) and NCPRS (global phenotype severity), and negative correlations with MVRD (MRI volumetric residuals), choline, and age. PC2 correlates negatively with *myo*‐inositol, further supporting its potential as a biomarker linked to disease state. (C) Hierarchical clustering dendrogram based on DEGs, classifying patients into distinct subgroups that align with clinical severity categories. *p < 0.05, **p < 0.01, ***p < 0.001.

To integrate metabolomic and transcriptomic data, we performed principal component analysis (PCA) using only differentially expressed genes between patients with mild and severe phenotypes and selected the metabolites that change significantly between control and patients to explore whether they were associated with disease severity by projecting them as supplementary variables (Figure 5). Along the first principal component (PC1), patient samples were separated by clinical severity. Supplementary variables contributing significantly to PC1 included age, MVRD, ICARS, NCPRS, and choline. PC2 was primarily associated with *myo*‐inositol, which stratified samples into one mild and two severe patient clusters (Figure 5). These findings suggest an association between intracellular *myo*‐inositol levels and clinical variability in PMM2‐CDG.

## 4 Discussion

Multi‐omics approaches provide a powerful framework for dissecting disease mechanisms in rare metabolic disorders, enhancing diagnostic yield and enabling pathway‐level interpretation and identification of potential therapeutic targets. These strategies generate data that inform the development of potential therapeutic readouts and clinical trial endpoints [17]. While each omics layer offers a distinct snapshot of biological processes distinguishing patients from healthy individuals, their integration allows a more comprehensive understanding of pathogenic mechanisms [18]. Importantly, combining molecular profiling with detailed phenotypic characterization enables clinical stratification to be incorporated into data interpretation, facilitating the identification of molecular signatures associated with disease severity [42].

Applying this integrative framework to PMM2‐CDG, our proteomic analysis identified 43 proteins with significantly altered abundance in patient‐derived fibroblasts. Although this number is lower than the 409 differentially expressed genes identified in our previous RNA‐seq study [17], the proteomic findings provide direct functional insight by capturing alterations at the protein level. Notably, proteomic data support the pathogenic mechanism of PMM2 variants by confirming their destabilizing effects at the protein level, thereby highlighting protein folding and stability as a potential therapeutic target [43]. The discrepancy between transcriptomic and proteomic datasets may reflect the technical limitations of mass spectrometry in detecting low‐abundance peptides, making it less sensitive than RNA‐seq for subtle changes. Moreover, mRNA levels do not always correlate with protein abundance due to post‐transcriptional regulatory mechanisms, including miRNA activity, protein degradation, and post‐translational modifications [44]. In the context of PMM2‐CDG, hypoglycosylation may further compromise protein folding, secretion, and stability, contributing to divergence between transcript and protein profiles. Together, these observations highlight the value of proteomics as a complementary layer to transcriptomic analysis, providing functional validation of disease mechanisms while underscoring the complexity of gene‐protein relationships in glycosylation disorders. In this regard, glycoproteomic approaches represent a logical extension of the present work and may offer additional resolution for identifying glycosylation‐dependent alterations in PMM2‐deficient cells [45].

One of the most prominent findings in PMM2‐CDG fibroblasts was the dysregulation of the retinoic acid synthesis pathway, characterized by significant downregulation of ALDH1A1, alongside upregulation of DHRS3, ALDH1A3, and ADH1B. Notably, AKR1B1 displayed discordant regulation, with increased protein levels despite decreased transcript abundance, possibly reflecting post‐transcriptional or post‐translational regulatory mechanisms. Similarly, although ALDH1A3 was consistently upregulated in the transcriptomic and proteomic datasets, western blot validation revealed substantial inter‐individual variability, suggesting a more complex regulatory profile. Possible explanations include post‐transcriptional regulation, protein instability, or the expression of transcript isoforms that do not yield stable protein products. In contrast, for ALDH1A1, we observed strong concordance across transcriptomic, proteomic, and validation datasets. The marked downregulation identified in the proteomic and transcriptomic screenings was confirmed by a consistent loss of protein expression in the larger validation cohort (Figure 2C,D and Figure S1), supporting ALDH1A1 as one of the most robust molecular alterations identified in this study. Importantly, although serum deprivation used for transcriptomic and proteomic analyses may influence metabolic pathways, the key finding regarding retinoic acid metabolism was independently validated under standard culture conditions by western blot analysis. The consistent reduction in ALDH1A1 protein levels under non‐synchronized conditions supports the conclusion that this alteration is not solely attributable to the synchronization protocol and likely reflects a disease‐associated molecular feature of PMM2‐CDG fibroblasts. ALDH1A1 belongs to a family of aldehyde dehydrogenases that catalyze the irreversible conversion of retinaldehyde to retinoic acid, the active form of vitamin A that regulates gene transcription via the retinoic acid receptor (RAR). RAR forms a heterodimer with the retinoid X receptor (RXR) and binds to specific DNA regions known as retinoic acid response elements (RAREs). Through this signalling axis, retinoic acid plays essential roles in embryonic development, including craniofacial and skeletal patterning, as well as in cell differentiation, immune regulation, and nervous system development [46, 47, 48]. Given that ALDH1A1 catalyzes a key step in retinoic acid biosynthesis, its consistent downregulation at both the mRNA and protein levels raises the possibility that altered retinoic acid pathway may contribute to some of the developmental manifestations observed in PMM2‐CDG and related glycosylation disorders (Table 1) [47, 49]. Future functional studies in animal models will be necessary to further elucidate the contribution of disrupted retinoic acid pathway to PMM2‐CDG pathogenesis and to assess its potential as a therapeutic target.

In addition to retinoic acid synthesis pathway, wound healing emerged as an enriched biological process, involving components of the Wnt signalling pathway and proteins regulating cell proliferation, migration, and extracellular matrix remodelling. These findings are consistent with our previous transcriptomic data [17] and may be functionally linked to altered vitamin A metabolism, as vitamin A deficiency is known to impair epithelialization and wound repair through reduced collagen synthesis and cross‐linking [50]. Such alterations may contribute to connective tissue manifestations commonly observed in PMM2‐CDG, including joint hyperlaxity and muscle weakness, which are associated with delayed motor development.

Metabolomic profiling using NMR identified seven significantly altered polar metabolites in PMM2‐CDG fibroblasts compared to controls. This targeted metabolic signature aligns with recent metabolomic data reported in a PMM2 zebrafish model and in peripheral blood mononuclear cells (PBMCs) from PMM2‐CDG patients [14, 51], while also revealing novel findings. Specifically, glutamine levels were elevated in patient‐derived fibroblasts, consistent with observations in the zebrafish model [14], whereas lysine and isoleucine were reduced. To further contextualize these findings at the systemic level, we extended our analysis to plasma amino acid profiles from 23 PMM2‐CDG patients. Despite inter‐individual variability, consistent patterns emerged: plasma aspartate and glutamate were significantly elevated, while asparagine, methionine, and cystine were reduced. Notably, glutamine levels showed opposite trends in plasma and fibroblasts—decreased in plasma but elevated intracellularly. To investigate whether intracellular glutamine accumulation could be explained by increased uptake, we measured glutamine levels in conditioned culture medium. However, glutamine concentrations in the medium were not reduced in PMM2‐CDG cultures, arguing against increased glutamine uptake as the primary explanation for the elevated intracellular levels. The discrepancy between plasma and fibroblast glutamine levels may instead reflect differences between systemic metabolism and cell‐autonomous metabolic adaptations in cultured cells. This discrepancy may reflect the fact that plasma glutamine concentrations are influenced by the metabolic activity of multiple tissues and organs, therefore representing systemic metabolic alterations that are not fully captured in cultured fibroblasts. Together, these data underscore the value of integrating cellular and systemic metabolomic profiling to capture disease heterogeneity and to complement findings from animal models.

The observed amino acid alterations may reflect increased turnover of misfolded or hypoglycosylated proteins, a known consequence of PMM2 deficiency. Beyond their structural roles, amino acids also function as key metabolic intermediates and signalling molecules [52]. For example, glutamine serves as a substrate for UDP‐GlcNAc synthesis through the hexosamine biosynthetic pathway (HBP), linking altered amino acid availability to downstream glycosylation‐related metabolism. Through interconnected metabolic routes, including the aspartate–glutamate shuttle, altered glutamine availability may influence the levels of other metabolites such as aspartate, asparagine, and succinate. In plasma, the combination of elevated glutamate and aspartate with reduced glutamine may reflect systemic metabolic adaptations to increased intracellular HBP activity. Reductions in isoleucine, proline, and lysine, which align with PBMC data, may point to defective protein turnover or mitochondrial dysfunction, a pathomechanism increasingly implicated in PMM2‐CDG and related glycosylation disorders [53].

In addition to amino acid dysregulation, we detected elevated levels of *myo*‐inositol, a metabolite derived from an alternative glucose‐6‐phosphate pathway. This finding aligns with prior MRI studies reporting increased *myo*‐inositol levels in the brains of PMM2‐CDG patients [54]. *Myo*‐inositol plays essential roles in phosphatidylinositol synthesis, membrane structure, second messenger signalling, and osmotic regulation, with particular relevance to liver and brain physiology [55]. Given the involvement of these organs in PMM2‐CDG, evaluating *myo*‐inositol levels in hepatic or neuronal models may be informative. Importantly, *myo*‐inositol can be quantified in multiple biological compartments, including plasma, urine, and brain tissue by MRI, supporting its potential translational relevance [56, 57]. Another glucose‐derived metabolite, sorbitol, has previously been proposed as a biomarker for PMM2‐CDG based on the correlation between urinary levels and disease severity [24]. In this context, future studies evaluating *myo*‐inositol levels in accessible patient samples and their association with clinical severity will be required to determine its potential utility as a biomarker.

Beyond its diagnostic potential, *myo*‐inositol has been reported to modulate endoplasmic reticulum (ER) homeostasis by facilitating ER membrane expansion and alleviating ER stress [58]. Although speculative in the context of PMM2‐CDG, these observations raise the possibility that altered *myo*‐inositol metabolism may reflect adaptive cellular responses to proteostatic stress. Future studies will be required to determine whether modulation of *myo*‐inositol levels could have therapeutic relevance.

We also found increased UDP‐GlcNAc levels in patient‐derived fibroblasts. This essential sugar donor for N‐glycosylation is synthesized via the HBP, whose first and rate‐limiting step is catalyzed by glutamine–fructose‐6‐phosphate amidotransferases 1 and 2 (GFPT1 and GFPT2), using fructose‐6‐phosphate and glutamine as substrates [41, 59]. GFPT1 is constitutively expressed and maintains basal HBP flux, whereas GFPT2 is an inducible isoform that responds to cellular stress and metabolic demands [59, 60]. In our multi‐omics datasets, increased UDP‐GlcNAc levels were accompanied by selective upregulation of GFPT2 at both the proteomic and transcriptomic levels, supporting the interpretation that HBP activity is actively upregulated rather than reflecting passive accumulation of glycosylation donors due to impaired PMM2‐dependent N‐glycosylation. In the context of PMM2 deficiency, redirection of glycolytic intermediates toward fructose‐6‐phosphate together with increased glutamine availability may contribute to enhanced HBP input. Furthermore, UDP‐GlcNAc serves as a substrate for O‐GlcNAcylation, a post‐translational modification that competes with phosphorylation and modulates key signalling pathways [61]. Whether increased UDP‐GlcNAc contributes directly to PMM2‐CDG pathogenesis or represents a compensatory mechanism remains uncertain. A recent multi‐omics study in PMM2‐deficient zebrafish linked N‐ and O‐glycosylation defects, raising the possibility that O‐glycosylation abnormalities play a role in disease progression [14]. Our current study provides support for these alterations in patient‐derived cells with diverse genetic backgrounds, providing critical evidence for this hypothesis.

Recent studies have shown that the HBP can be transcriptionally regulated by XBP1s, the spliced and active form of the transcription factor XBP1, which is induced during the Unfolded Protein Response (UPR) [14]. However, neither XBP1 nor other canonical UPR genes were upregulated in our transcriptomic dataset, consistent with previous reports in PMM2‐CDG fibroblasts [62]. This suggests that the UPR is not prominently activated in patient fibroblasts, unlike in PMM2 zebrafish models.

An alternative hypothesis may involve inflammatory or stress‐responsive transcriptional programs. GFPT2 has been reported in other pathological contexts to be regulated by NF‐κB, a transcription factor activated by inflammatory signals and ER stress [60]. NF‐κB also regulates genes involved in prostaglandin synthesis, including *PTGS2* (COX‐2), which we previously identified as dysregulated in PMM2‐CDG in an independent transcriptomic study [17]. Although the present study does not directly assess inflammatory pathway activation or establish causality, these observations raise the possibility that dysregulated HBP activity and inflammatory signaling may converge on shared upstream regulators in PMM2‐CDG. Future studies will be required to directly evaluate NF‐κB activity and to determine whether modulation of HBP components, including GFPT2, influences inflammatory outputs or cellular homeostasis in PMM2‐CDG models. Such work will be essential to clarify whether HBP activation represents a compensatory adaptation or contributes to disease pathophysiology. Finally, the metabolomic analysis combined with clinical severity stratification revealed a clear metabolic distinction between milder (LOW) and more severe (HIGH) PMM2‐CDG phenotypes, as captured by principal component analysis (PCA). Metabolites such as UDP‐GlcNAc and short‐chain fatty acids were relatively enriched in the HIGH group, consistent with alterations in the HBP and lipid metabolism. These patterns are consistent with broader metabolic rewiring and adaptive responses to sustained cellular stress. In contrast, choline showed higher relative abundance in the LOW group. Choline plays a key role in membrane phospholipid synthesis and methyl group metabolism, and it also has anti‐inflammatory properties through its involvement in the cholinergic anti‐inflammatory pathway [63]. Higher choline levels in milder cases may therefore reflect a more stable cellular state, possibly associated with reduced cellular stress or inflammatory signaling.

In summary, integrating transcriptomic, proteomic, and metabolomic data in PMM2‐CDG reveals widespread molecular alterations across multiple biological layers. Consistent signatures—including elevated *myo*‐inositol, dysregulated retinoic acid synthesis pathway, and altered UDP‐sugar levels—provide new insights into disease‐associated metabolic and molecular alterations. These results underscore the value of combining cellular and systemic omics approaches to uncover disease mechanisms and generate hypotheses for future biomarker validation studies and therapeutic development in PMM2‐CDG and related glycosylation disorders.

## Author Contributions

**Diana Gallego:** conceptualization, methodology, validation, formal analysis, investigation, writing – original draft, visualization. **Giuseppina Andreotti:** methodology, validation, formal analysis, investigation. **Maria Monticelli:** methodology, validation, formal analysis, investigation. **Debora Paris:** conceptualization, methodology, validation, formal analysis, investigation, funding acquisition. **Arturo Martín‐Martínez:** methodology, validation, formal analysis, investigation. **Alejandra Gámez:** formal analysis, investigation. **Mercedes Serrano:** resource, formal analysis, investigation. **José Córdoba‐Caballero:** methodology, validation, formal analysis. **Pedro Seoane:** conceptualization, software, methodology, validation, formal analysis, investigation. **Juan A.G. Ranea:** conceptualization, supervision. **Belén Pérez:** conceptualization, resources, writing – original draft, methodology, review and editing final draft, supervision, visualization, project administration, funding acquisition.

## Funding

This work was supported by the Carlos III Health Research Institute (ISCIII) and FEDER under grant numbers PI22/00699, PI25/01398 and XXII Concurso Nacional para la adjudicación de Ayudas a la investigación en Ciencias de la Vida y de la Materia, 2024 de la Fundación Ramón Areces awarded to B.P. It was also partially funded through the Accordo per la coesione della Regione Campania—Promozione di progetti di ricerca, sviluppo sperimentale e innovazione collaborativi nel campo delle malattie rare, Intervento no. 28 (CUP: B83C25000740002), awarded to G.A. G.A. also acknowledges support from the CNR Short‐Term Mobility Program (2019).

## Ethics Statement

The study was conducted in accordance with the Declaration of Helsinki and approved by the Ethics Committee of Universidad Autónoma de Madrid (CEI‐1029‐2655). Before analysis, written informed consent for genetic testing was obtained from the patients or their legal guardians.

## Consent

All authors have read the manuscript and have given their consent for publication.

## Conflicts of Interest

The authors declare no conflicts of interest.

## Supporting information


**Figure S1:** Representative western blot showing ALDH1A1 and ALDH1A3 protein levels in control and PMM2‐CDG fibroblasts. GAPDH was used as a loading control.

**Figure S2:** Glutamine levels in conditioned medium and fibroblast lysates. Glutamine concentrations were measured by ion‐exchange chromatography in conditioned culture medium (A) and fibroblast lysates (B) from control and PMM2‐CDG fibroblasts. Medium values were normalized to cell number and intracellular values were normalized to protein concentration. No statistically significant differences were observed between groups.

**Table S1:** Patient data.

**Table S2:** List of oligonucleotides for RT‐qPCR studies.

**Table S3:** Proteins exhibiting statistically significant abundance changes in PMM2‐CDG fibroblasts relative to controls.

**Table S4:** Metabolomics—raw data.
**Table S4:** Metabolomics—peak assignment.
**Table S4:** Metabolomics—statistics.

## Data Availability

The data that support the findings of this study are available on request from the corresponding author. The data are not publicly available due to privacy or ethical restrictions.
