# Supplementary material for: Proteo‐Metabolomic Profiling of PMM2‐CDG Reveals Dysregulation of Retinoic Acid Synthesis, Myo‐Inositol, and the Hexosamine Pathway
Source: J Inherit Metab Dis. 2026 Aug 13;49(5):e70233. doi: 10.1002/jimd.70233 (PMC13471400; doi:10.1002/jimd.70233)
Supplement: Supplementary file 1 [file JIMD-49-0-s002.docx]

**Supplementary Table 2. List of oligonucleotides for RT-qPCR studies**

| **Primer Name** | **Sequence** |
| --- | --- |
| *ALDH1A1* Fw | ACTTACCTGTCCTACTCACCGA |
| *ALDH1A1* Rv | TCCTCCTTATCTCCTTCTTCTACC |
| *ADH1B* Fw | CAACCCTCAAGACTACAAGAAACCC |
| *ADH1B* Rv | GACAACATAACAGGGAAGCCATCAT |
| *ALDH1A3* Fw | AAATCTCGACAAAGCCCTGAAGTTG |
| *ALDH1A3* Rv | GCCAAAGCGTATTCACCTAGTTCTC |
| *DHRS3* Fw | AATGCATGCCCTCGTTATCTTGAAA |
| *DHRS3* Rv | CTATGTCCGCCCTTTGAAAGTGTTC |
| *AKR1B1* Fw | ATCTCCAACTTCAACCATCTCCAGG |
| *AKR1B1* Rv | ATATGGGTGGCACTCAATCTGGTTA |
| *GFPT2* Fw | GCGGAATCTTTGCCTACATGAACTA |
| *GFPT2* Rv | ACTTCGTGATTATTCCCATCGATCG |
| *GUSB* Fw | AGAGTGGTGCTGAGGATTGG |
| *GUSB* Rv | CCCTCATGCTCTAGCGTGTC |
