# Supplementary material for: Proteo‐Metabolomic Profiling of PMM2‐CDG Reveals Dysregulation of Retinoic Acid Synthesis, Myo‐Inositol, and the Hexosamine Pathway
Source: J Inherit Metab Dis. 2026 Aug 13;49(5):e70233. doi: 10.1002/jimd.70233 (PMC13471400; doi:10.1002/jimd.70233)
Supplement: Supplementary file 2 [file JIMD-49-0-s005.docx]

**Supplementary Table 1: Patient data**

| **Patient** | **Pathogenic variants (NM_000303.3)** | **Sex** | **Age at diagnosis** | **PMM2 activity in fibroblasts** | **Phenotype** | **Omic layer** |
| --- | --- | --- | --- | --- | --- | --- |
| **P1** | c.710C>G p.(Thr237Arg)^†^  c.710C>G p.(Thr237Arg)^†^ | M | 4.5y | 29 % | Mild | Metabolic analysis |
| **P2** | c.256-1G>T p.?  c.95T>G p.(Leu32Arg)^†^ | F | 3.5y | 26 % | Mild | Metabolic analysis |
| **P3** | c.422G>A p.(Arg141His)  c.722G>C p.(Cys241Ser)^†^ | M | 9y | 34 % | Mild | Metabolic analysis |
| **P4** | c.422G>A p.(Arg141His)  c.95T>G p.(Leu32Arg)^†^ | M | 4y 1m | - | Mild | Metabolic analysis |
| **P5** | c.523+3A>G p.?  c.484C>T p.(Arg162Trp)^†^ | M | - | - | Mild | Metabolic analysis |
| **P6** | c.640-9T>G p.?  c.710C>T p.(Thr237Met)^†^ | F | 1y | 12 % | Moderate | Proteomic analysis/ Metabolomic analysis |
| **P7** | c.422G>A p.(Arg141His)  c.484C>T p.(Arg162Trp)^†^ | F | 11y | 35 % | Unclassified | Proteomic analysis/ Metabolomic analysis |
| **P8** | c.422G>A p.(Arg141His)  c.710C>T p.(Thr237Met)^†^ | M | 5y | 20 % | Severe | Metabolic analysis |
| **P9** | c.422G>A p.(Arg141His)  c.193G>T p.(Asp65Tyr)^†^ | M | 1y 1m | 25 % | Severe | Proteomic analysis/ Metabolomic analysis |
| **P10** | c.422G>A p.(Arg141His)  c.191A>G p.(Tyr64Cys)^†^ | M | 4m 27d | 19 % | Severe | Metabolic analysis |
| **P11** | c.620T>C p.(Phe207Ser)^†^  c.415G>A p.(Glu139Lys)^†^ | M | 6m 18d | 10.30 % | Severe | Metabolic analysis |
| **P12** | c.338C>T p.(Pro113Leu)^†^  c.710C>A p.(Thr237Lys)^†^ | M | <5y | - | Severe | Metabolic analysis |
